# Case Report: A case of incidentally discovered isolated gastric plasmacytoma

**DOI:** 10.3389/fonc.2026.1899646

**Published:** 2026-07-07

**Authors:** Ya-nan Zhang, Qing-xin Li, Lin-chong Ma, Yong-tai Jin, Wen-qi Li, Jin-chen Du

**Affiliations:** 1 Department of High Altitude Medicine, 940th Hospital of Joint Logistics Support Force, Chinese People’s Liberation Army, Lanzhou, Gansu, China; 2 Department of Thoracic Surgery, 940th Hospital of Joint Logistics Support Force, Chinese People’s Liberation Army, Lanzhou, Gansu, China; 3 First Clinical College, Gansu University of Traditional Chinese Medicine, Lanzhou, Gansu, China

**Keywords:** endoscopic resection, extramedullary plasmacytoma, gastric plasmacytoma, immunohistochemistry, pathological diagnosis, solitary plasmacytoma

## Abstract

Extramedullary plasmacytoma (EMP) is a rare plasma cell malignancy, accounting for approximately 3% of all plasma cell neoplasms. Gastric plasmacytoma, a distinct subtype of EMP arising from B lymphocytes, is extremely uncommon, representing roughly 2% of EMP cases, and lacks specific clinical manifestations. Herein, we report a 53-year-old male presenting with persistent acid reflux and heartburn. Gastroscopy identified a flat, elevated lesion in the gastric body. Endoscopic mucosal resection (EMR) was performed to obtain the lesion specimen. The diagnosis of primary solitary gastric plasmacytoma was confirmed via histopathology, immunohistochemistry, bone marrow aspiration, serum and urine free light chain assays, and positron emission tomography–computed tomography (PET-CT). We further review the clinical features, diagnostic approaches, therapeutic strategies, and prognosis of this disease, aiming to provide a reference for the diagnosis and management of rare gastric plasma cell tumors in clinical practice.

## 1 Introduction

Extramedullary plasmacytoma (EMP) is a rare malignant neoplasm characterized histopathologically by the infiltration of plasma cells at various maturation stages and the presence of monoclonal features. This disease has a marked male predominance, with males affected far more frequently than females (1). Extramedullary plasmacytoma may arise in any lymphoid tissue-containing organ, most frequently in the head and neck region; the upper respiratory tract and oral cavity account for 80%–90% of all cases (2–4). Primary gastric plasmacytoma (PGP) constitutes less than 5% of all EMP cases. PGP manifests insidiously, primarily with nonspecific upper gastrointestinal symptoms. Endoscopically, it exhibits diverse morphologies, classified as polypoid, ulcerative, nodular, or diffuse infiltrative types (5). In the early stage, PGP is often misdiagnosed as a benign lesion or gastric carcinoma. Currently, the diagnosis of PGP relies on histopathology and immunohistochemistry, with strict exclusion of multiple myeloma (MM) and extramedullary lesions at other sites. No uniform treatment guidelines have been established. For early localized disease, surgical or endoscopic resection is the first-line therapy, while advanced cases require adjuvant chemoradiotherapy. Globally, large-scale prospective clinical studies specifically focusing on primary gastric plasmacytoma remain scarce. Although several retrospective cohort studies on extramedullary plasmacytoma have been published in Europe and North America, relevant large-sample data are still insufficient worldwide. Consistent with the current situation in other countries, high-quality large-scale clinical investigations are also lacking in China. Due to its rarity and insufficient large-scale clinical data domestically, the etiology, differential diagnostic criteria and long-term prognosis of PGP have not been fully elucidatedPG. Here, we report a case of primary solitary gastric plasmacytoma confirmed by endoscopic resection, with MM completely excluded, to enhance clinical awareness and reduce missed or incorrect diagnoses.

## 2 Case presentation

A 53-year-old male presented with a 6-month history of acid reflux, heartburn, and dull epigastric pain that occurred intermittently three to four times per week. His symptoms worsened over the past month, accompanied by intermittent melena. The patient reported symptomatic improvement with omeprazole (20 mg twice daily) but recurrence after treatment discontinuation. The patient had no relevant family history of hematological malignancies, gastrointestinal tumors or other hereditary diseases. His past medical history was unremarkable except for chronic gastritis diagnosed 5 years earlier. He denied hypertension, diabetes, cardiovascular diseases or surgical history. The patient had a long-term heavy drinking history for more than 20 years, consuming approximately 150–200 mL of distilled spirits daily; he had no history of tobacco use. Prior to this admission, he had intermittently taken oral proton pump inhibitors for epigastric discomfort without regular standardized treatment. On physical examination, no superficial lymphadenopathy was palpable. Cardiopulmonary auscultation was unremarkable. The abdomen was flat and soft, with mild tenderness below the xiphoid process and no rebound tenderness or muscular guarding. The liver and spleen were non-palpable, shifting dullness was absent, and bowel sounds were normal. Chest computed tomography (CT) revealed no abnormalities in the lungs or mediastinum. Laboratory tests showed urine occult blood was positive (2+), 24-hour urinary total protein of 144 mg, 14C-UBT: DPM = 186 (>100) and positive fecal occult blood. Liver and renal function, electrolytes, lactate dehydrogenase (LDH), and β2-microglobulin were all within normal ranges. Complete blood count showed hemoglobin of 153 g/L and platelet count of 170 × 10^9^/L.

## 3 Diagnostic assessment

**Figure 1:**
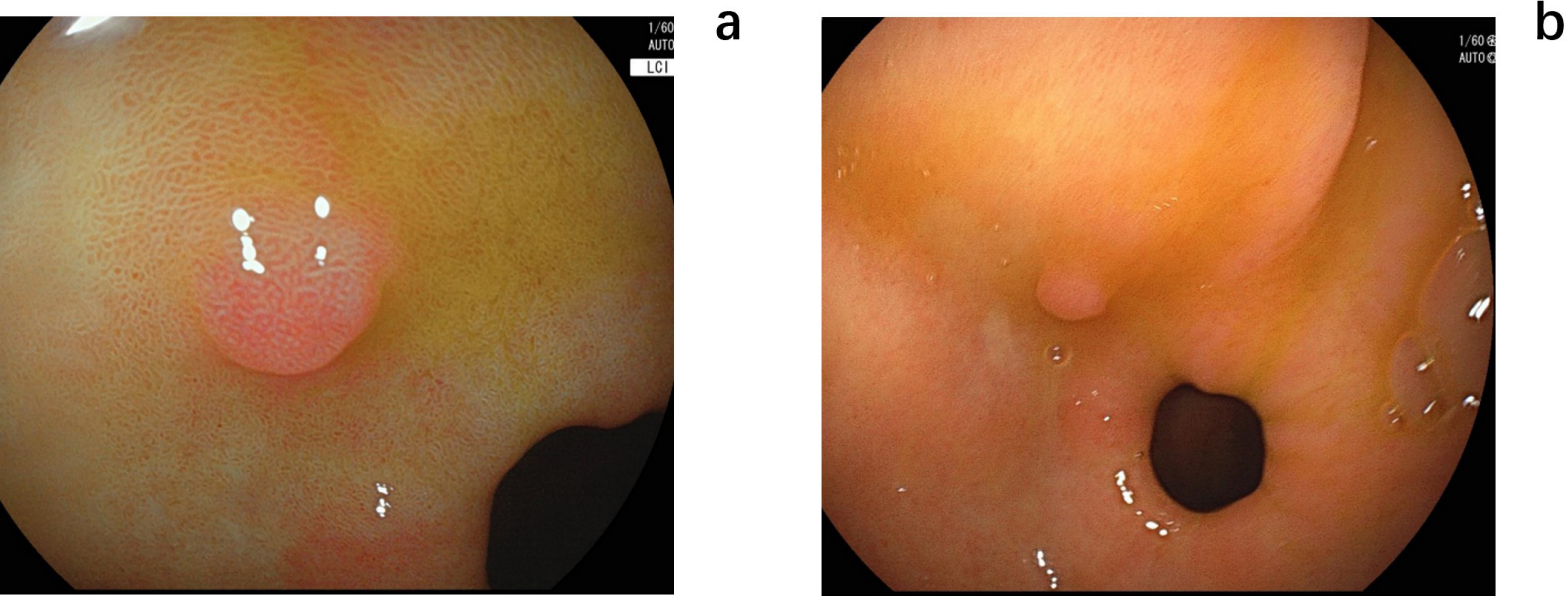
Gastroscopic image. **(a)** A flat elevation (0.4 cm × 0.5 cm) on the posterior wall of the lower body of the stomach; broad-based with a smooth surface; **(b)** A hemispherical elevation (0.4 cm × 0.4 cm) in the antrum near the pylorus; pedunculated with a smooth surface.

The initial clinical diagnosis was chronic gastritis. Gastroscopy revealed a flat, smooth, whitish lesion (0.4–0.5 cm) on the posterior wall of the gastric lower body (**Figure 1a**) and a hemispherical elevation (0.4 cm × 0.4 cm) with a smooth white surface in the gastric antrum (**Figure 1b**). Endoscopic mucosal resection (EMR) was performed for both lesions, achieving gross total resection.

**Figure 2:**
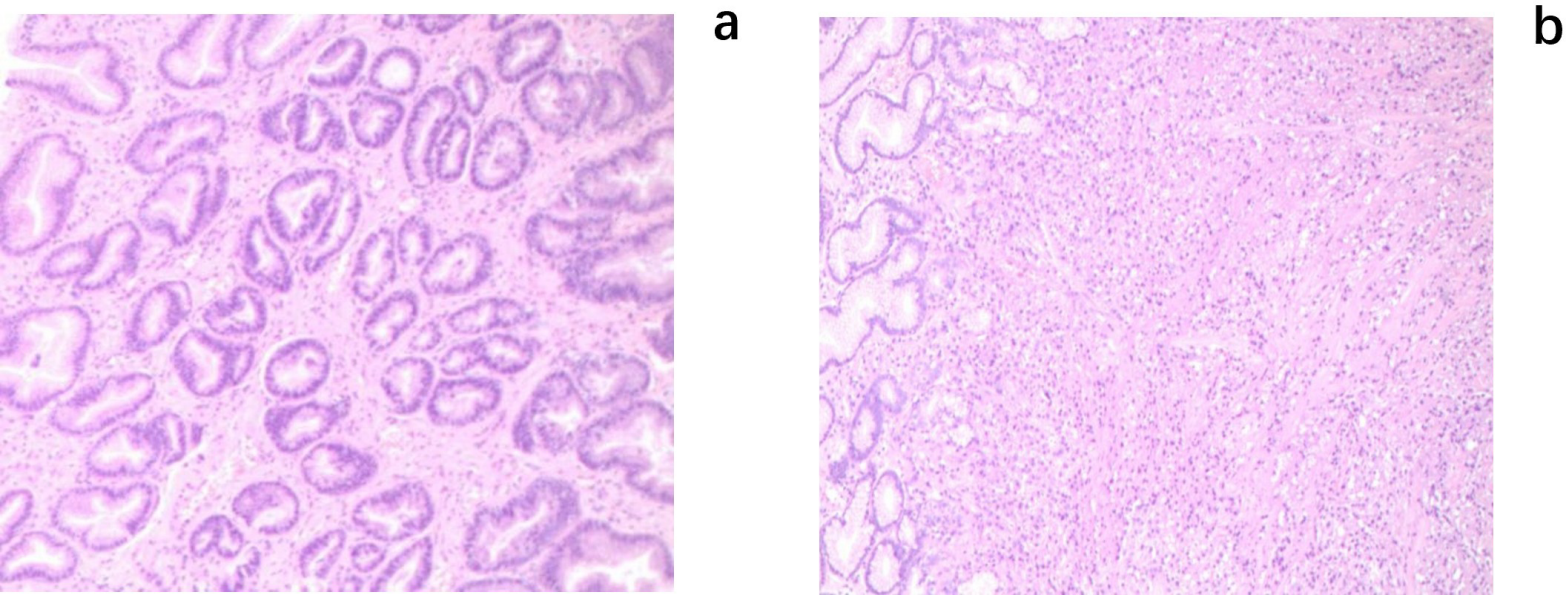
Histopathological HE stain (×200). **(a)** Diffuse infiltration of numerous plasma cells in the lamina propria of the gastric body lesion, with mild atypia; **(b)** Hyperplastic polyp of the gastric antrum, with regular glands and no atypical cells.

**Figure 3:**
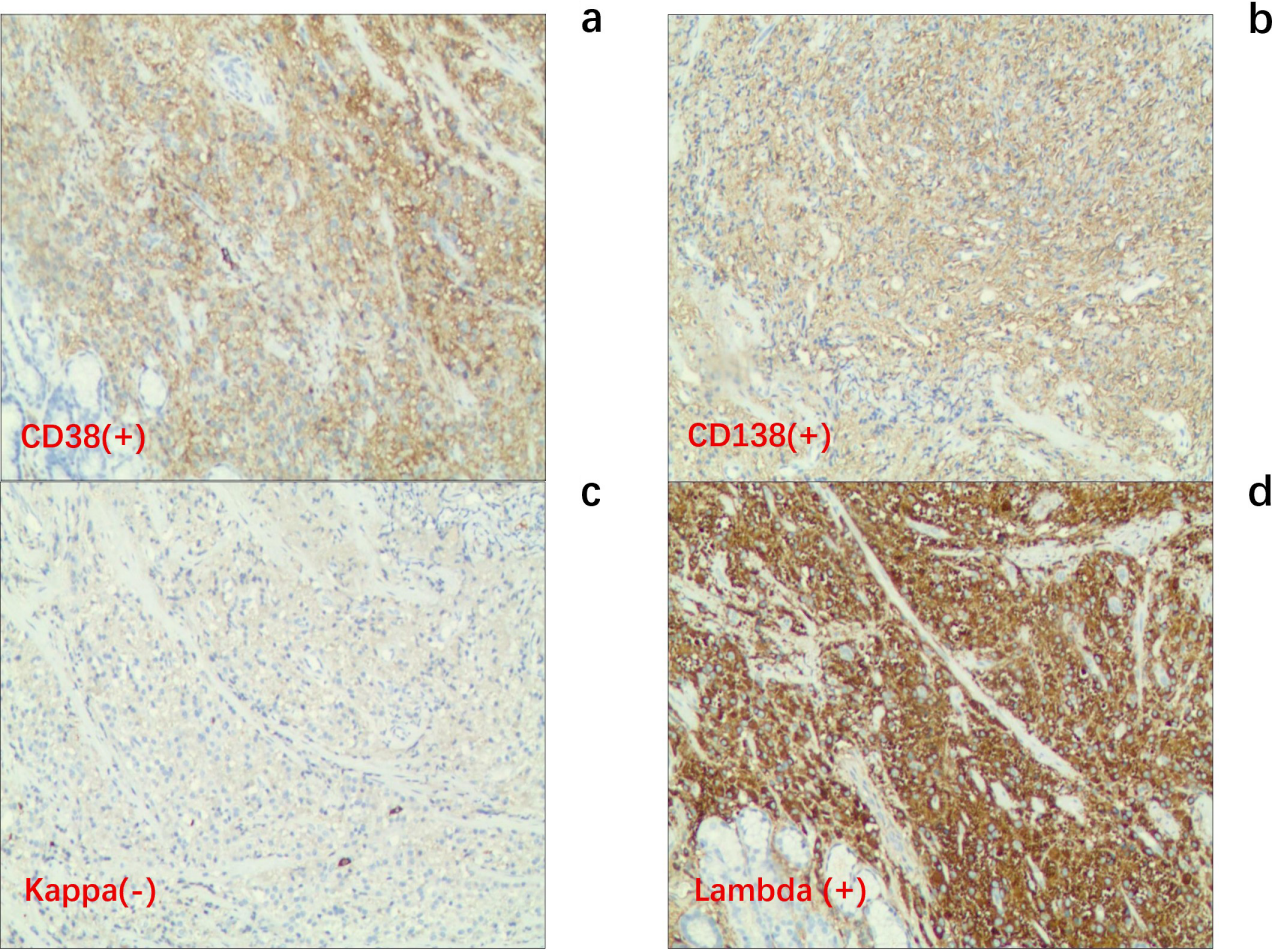
Immunohistochemical staining results of gastric lesion tissue (×200). **(a)** Diffuse, strong positive expression of CD38; **(b)** Diffuse, strong positive expression of CD138; **(c)** Negative expression of kappa light chains; **(d)** Diffuse, strong positive expression of lambda light chains, suggesting light-chain-restricted expression.

Postoperative histopathology demonstrated diffuse infiltration of numerous monoclonal plasma cells with mild atypia in the lamina propria of the gastric body lesion, with adjacent gastric glands compressed (**Figure 2a**). The gastric antrum, however, contains a normal hyperplastic polyp (**Figure 2b**). The lesion was strictly confined to the mucosa and lamina propria, with no extension into the muscularis mucosa or deeper layers. No lymphovascular invasion or perineural invasion was observed in the specimen. Histological examination confirmed negative horizontal resection margins and negative vertical resection margins, indicating pathological complete resection. Immunohistochemical analysis of the gastric body lesion showed: CKp (-), CK8/18(-), CDX2(-), Villin(-), CD68(-), SOX10(-), S100(-), Ki67 ≈ 3%, CD138(+) (**Figure 3a**), CD38(+) (**Figure 3b**), Kappa(-) (**Figure 3c**), Lambda(+) (**Figure 3d**).

To exclude MM and differentiate from primary low-grade gastric tumors, the patient underwent serum and urine free light chain testing, PET-CT, bone marrow aspiration, and serum protein electrophoresis. Urine free light chains: κ 59.27 mg/L, λ 7.73 mg/L (κ/λ = 7.67); serum free light chains: κ 17.53 mg/L, λ 18.70 mg/L (κ/λ = 0.94). PET-CT detected no metastatic lesions. Bone marrow aspiration showed normal cellularity, with plasma cells accounting for 0.36% (normal range <5%) and no clonal or immature plasma cells identified. Serum protein electrophoresis revealed no M-protein. Notably, the abnormal κ/λ ratio in urine was attributed to monoclonal light chains secreted locally by the gastric lesion. The normal serum free light chain ratio, negative M-protein and normal bone marrow plasma cell percentage collectively ruled out systemic plasma cell disorders such as multiple myeloma. Based on clinical manifestations, imaging findings, and pathological results, the final diagnosis was primary solitary gastric plasmacytoma.

## 4 Discussion

PGP predominantly affects middle-aged and elderly males, with a median age at diagnosis of 50–60 years. Clinical symptoms are nonspecific, including epigastric pain, abdominal distension, acid reflux, and anorexia (6, 7); some patients present initially with gastrointestinal bleeding, while a small proportion are asymptomatic. Consistent with previous reports, our patient presented primarily with acid reflux and heartburn, accompanied by intermittent melena. The initial misdiagnosis as chronic gastritis resulted from the substantial overlap between PGP symptoms and those of common gastric disorders, combined with limited clinical vigilance toward rare plasma cell tumors. The pathogenesis of PGP remains unclear but is thought to be associated with chronic inflammatory stimulation, immune dysfunction, Helicobacter pylori infection, and long-term alcohol consumption. Consistent with these risk factors, the present patient had positive Helicobacter pylori status and a long history of heavy alcohol drinking. Persistent chronic inflammation of the gastric mucosa may induce aberrant B lymphocyte proliferation, potentially representing a key contributing factor (8).

### 4.1 Diagnosis

PGP is rare and lacks specific clinical manifestations. Imaging findings typically show homogeneous enhancement and focal thickening of the gastric wall; early PGP may also manifest as a well-circumscribed intraluminal or exophytic mass. Definitive diagnosis relies on histopathological examination and immunohistochemistry. Biopsy specimens demonstrate diffuse abnormal plasma cell proliferation with cellular atypia. Immunohistochemistry is critical for differentiation, typically showing positivity for CD38, CD138, and CD79a, and negativity for CD20, CD3, and carcinoembryonic antigen (CEA) (9–11). Additionally, serum protein electrophoresis, immunofixation electrophoresis, bone marrow aspiration, and imaging studies are required to evaluate disease extent (12).

### 4.2 Differential diagnosis

Multiple myeloma: Characterized by ≥10% plasma cells in the bone marrow, associated with bone pain, osteolytic lesions, and elevated M-protein levels; excluded in this case given normal bone marrow plasma cell proportion and absence of bone destruction.

Gastric lymphoma: Typically CD20-positive and CD138-negative.

Poorly differentiated gastric carcinoma: Usually cytokeratin (CK)-positive and CEA-positive.

Hyperplastic polyp: No atypical plasma cell infiltration.

### 4.3 Treatment and prognosis

Currently, no standardized treatment protocols exist for gastric plasmacytoma, with management involving a multimodal approach combining surgery, endoscopic resection, radiotherapy, and chemotherapy. For early localized disease, surgical resection or endoscopic resection is widely adopted as radical treatment for superficial mucosal lesions (13). Notably, solitary extramedullary plasmacytoma is highly radiosensitive, and radiotherapy serves as a vital local therapeutic option across disease stages. Radiotherapy is recommended for patients with unresectable lesions, positive resection margins, or high-risk pathological features, and it achieves excellent local disease control rates for extramedullary plasmacytoma. Multiple clinical studies have demonstrated favorable long-term local control and survival outcomes after radiotherapy for this disease (14–16). Chemotherapy is primarily administered as adjuvant therapy postoperatively or for advanced/metastatic disease, effectively inhibiting abnormal plasma cell proliferation (17). Furthermore, Helicobacter pylori eradication therapy may serve as first-line adjuvant treatment for infected patients (18, 19).

In this case, after confirming positive Helicobacter pylori infection, we administered the standard quadruple eradication regimen: esomeprazole 20 mg twice daily, amoxicillin 1000 mg twice daily, clarithromycin 500 mg twice daily, and bismuth potassium citrate 220 mg twice daily. The treatment course lasted 14 days. Four weeks after completion of the regimen, a repeat ^14^C-urea breath test was performed, and the result turned negative, confirming successful eradication.

The prognosis of gastric plasmacytoma is slightly poorer than that of extramedullary plasmacytoma at other sites but better than that of multiple myeloma, correlating with disease extent, treatment timing, therapeutic regimen, and patient general condition (5). Early diagnosis and prompt comprehensive therapy significantly improve survival. Studies report 5-year survival rates exceeding 70% in patients with early gastric plasmacytoma following aggressive treatment, with some achieving clinical remission (20).

This patient had stage I solitary disease confined to mucosa and lamina propria, with complete pathological resection via EMR and successful Helicobacter pylori eradication. At present, the patient has completed 3 months of regular follow-up, and no signs of local recurrence, distant metastasis or new clinical symptoms have been observed. Given the limited follow-up duration, we cannot draw definitive conclusions regarding long-term prognosis. Long-term continuous follow-up is still required to monitor potential recurrence.

## Data Availability

The original contributions presented in the study are included in the article/supplementary material. Further inquiries can be directed to the corresponding author.
